# C8-Substituted Imidazotetrazine Analogs Overcome Temozolomide Resistance by Inducing DNA Adducts and DNA Damage

**DOI:** 10.3389/fonc.2019.00485

**Published:** 2019-06-11

**Authors:** Zhikuan Yang, Danping Wei, Xiaoli Dai, Malcolm F. G. Stevens, Tracey D. Bradshaw, Ying Luo, Jihong Zhang

**Affiliations:** ^1^Medical School, Kunming University of Science and Technology, Kunming, China; ^2^Centre for Biomolecular Sciences, University of Nottingham, Nottingham, United Kingdom

**Keywords:** glioblastoma, colorectal carcinoma, *O6*-methylguanine-DNA methyltransferase, apoptosis, DNA adducts

## Abstract

Temozolomide (TMZ) is the standard of care chemotherapeutic agent used in the treatment of glioblastoma multiforme. Cytotoxic *O6*-methylguaine lesions formed by TMZ are repaired by *O6*-methyl-guanine DNA methyltransferase (MGMT), a DNA repair protein that removes alkyl groups located at the *O6*-position of guanine. Response to TMZ requires low MGMT expression and functional mismatch repair. Resistance to TMZ conferred by MGMT, and tolerance to *O6*-methylguanine lesions conferred by deficient MMR severely limit TMZ clinical applications. Therefore, development of new TMZ derivatives that can overcome TMZ-resistance is urgent. In this study, we investigated the anti-tumor mechanism of action of two novel TMZ analogs: C8-imidazolyl (377) and C8-methylimidazole (465) tetrazines. We found that analogs 377 and 465 display good anticancer activity against MGMT-overexpressing glioma T98G and MMR deficient colorectal carcinoma HCT116 cell lines with IC_50_ value of 62.50, 44.23, 33.09, and 25.37 μM, respectively. Analogs induce cell cycle arrest at G2/M, DNA double strand break damage and apoptosis irrespective of MGMT and MMR status. It was established that analog 377, similar to TMZ, is able to ring-open and hydrolyze under physiological conditions, and its intermediate product is more stable than MTIC. Moreover, DNA adducts of 377 with calf thymus DNA were identified: *N7*-methylguanine, *O6*-methylguanine, *N3*-methyladenine, *N3*-methylthymine, and *N3*-methylcytidine deoxynucleotides. We conclude that C8 analogs of TMZ share a mechanism of action similar to TMZ and are able to methylate DNA generating *O6*-methylguanine adducts, but unlike TMZ are able at least in part to thwart MGMT- and MMR-mediated resistance.

## Introduction

Temozolomide (TMZ), an oral alkylating agent, has been administered in conjunction with radiation as the standard of care for glioblastoma multiforme (GBM) treatment (1). TMZ is an imidazotetrazine prodrug that is able to cross the blood–brain barrier (BBB) (2). Under normal physiological conditions, TMZ spontaneously hydrolyzes to form the active intermediate 3-methyl-(triazen-1-yl) imidazole-4-carboxamide (MTIC). MTIC rapidly breaks down to form the reactive methyldiazonium ion (diazomethane) which reacts with nucleophilic groups on DNA, resulting in DNA methylation (3). Approximately 70% of the methyl groups are located on the *N7* of guanine (*N7*-G), 10% on *N3*-adenine (*N3*-A), and 5% at *O6*-guanine (*O6*-G) sites (2, 3). Interestingly, TMZ exerts cytotoxicity mainly through O6-methylguanosine (*O6*-MeG) which triggers futile cycles of mismatch repair (MMR), stalled replication forks and lethal DNA double-strand breaks, while *N7*- and *N3*-methyl purines are rapidly repaired by base excision repair (BER) (4, 5). However, *O6*-MeG lesions can be repaired by *O6*-methyl-guanine DNA methyltransferase (MGMT), a DNA repair protein that removes alkyl groups located at the *O6*-position of guanine (6). Thus, response to TMZ requires low MGMT expression and intact MMR (7, 8).

Resistance to TMZ conferred by MGMT and tolerance to *O6*-MeG in the presence of defective MMR limits TMZ clinical applications. Therefore, strategies have been devised to reduce resistance and enhance response to TMZ, including inhibition of DNA repair mechanisms which contribute to TMZ resistance. For example, combining MGMT inhibitors, which consume MGMT and prevent *O6*-MeG repair, with TMZ sensitizes tumors to TMZ treatment (9). However, myelosuppression limits the use of MGMT inhibitors and alkylating agent combination chemotherapy (10). Other DNA repair (BER) protein inhibitors have been used in combination with TMZ to treat cancer, such as poly ADP-ribose polymerase (PARP) inhibitors. Although PARP inhibitors (in combination with TMZ) are better tolerated than MGMT inhibitors, myelosuppression, and liver toxicity still cause clinical concern (11, 12).

**Figure 1:**
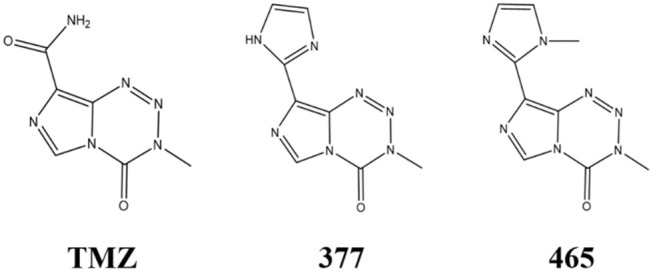
Chemical structures of TMZ, 377, and 465.

An alternative strategy is the development of TMZ analogs which can overcome TMZ-resistance. A series of TMZ derivatives has been synthesized and the anticancer activity was investigated *in vitro*. N3-substituted TMZ analogs were developed and several derivatives exhibited an activity against TMZ-resistant tumor cells *in vitro* (13–16). In contrast, C8-substituted TMZ analogs have rarely been reported and compounds mechanisms of action remain unclear (17). In this study, the anti-tumor effects of two novel C8-substituted TMZ derivatives have been investigated in TMZ-resistant tumor models *in vitro*. The C8 carboxamide was replaced by imidazolyl (377) and methylimidazole (465) groups, respectively (Figure 1). 377 and 465 analogs demonstrated good anticancer activity against MGMT overexpressing glioma T98G and MMR deficient colorectal carcinoma HCT116 cell lines. Moreover, 377 and 465 were able to arrest the cell cycle at G2/M, evoke DNA damage, and trigger apoptosis in T98G GBM and HCT116 CRC cells, irrespective of MGMT and MMR status. The mechanism by which analog 377 exacts DNA damage, and specific DNA lesions produced have been identified. Like TMZ, analog 377 ring-opens and hydrolyzes under physiological conditions leading to DNA adducts formation *N7*-methylguanine (*N7*-MeG), *O6*-methylguanine (*O6*-MeG), *N3*-methyladenine (*N3*-MeA), *N3*-methylthymine (*N3*-MeT), and *N3*-methylcytidine (*N3*-MeC) deoxynucleotides.

## Materials and Methods

### Chemicals

TMZ was provided by Schering-Plow Research Institute (Kenilworth, NJ, USA). New analogs, synthesized at Pharminox Ltd, BioCity, Nottingham, UK, were prepared as 100 mM stock solutions in dimethyl sulfoxide (DMSO) and stored at −20°C. Reagents, unless specified otherwise, originated from Sigma-Aldrich Ltd.

### Cell Lines and Culture Conditions

SNB19V (vector) and isogenic MGMT-transfected SNB19M human GBM cell lines were provided by Schering-Plow. Cells were cultured in RPMI 1640 medium supplemented with 2% glutamine, 1% non-essential amino acids, 50 μg/ml gentamicin, 400 g/ml G418 (geneticin), and 10% fetal bovine serum (FBS). HCT116 (hMLH1-) and HT29 CRC cells obtained from the American Type Culture Collection (ATCC), were maintained in RPMI 1640 medium supplemented with 10% FBS. T98G GBM cells (sourced from the ATCC) were grown in DMEM medium supplemented with 10% FBS. Cells were incubated in a humidified atmosphere of 95% air and 5% CO_2_ at 37°C.

### MTT Assay

Cells were seeded into wells at a density of 4 ×10^3^ per well in 96-well plates and allowed to attach overnight. TMZ, 377, or 465 dilutions were prepared in culture medium from 100 mM stock solutions and final well-concentrations between 0.1 and 1,000 μM were achieved (*n* = 3). Following 3 days of incubation (37°C, 5% CO_2_). Sterile-filtered 3-(4,5-dimethylthiazol-2-yl)-2,5-diphenyltetrazolium bromide (MTT) (20 μl; 5 mg/ml in phosphate buffered saline) was added to each well (final concentration 0.4 mg/ml). Plates were re-incubated for 4 h allowing metabolism of MTT by viable cells to insoluble formazan crystals. Medium and unconverted MTT were aspirated and DMSO (150 μl) was added to each well to ensure complete formazan solubilization, absorbance was read on a BioTek Synergy H1 microplate reader (490 nm). Compounds concentrations causing 50% inhibition (IC_50_) values were calculated by interpolation.

### Clonogenic Assay

Exponentially growing cells were seeded in triplicate at a density of 300 cells per well in 6-well plates and allowed to attach overnight before introduction of TMZ (final concentrations 5, 10, 100, 500, and 1,000 μM), 377 (final concentrations 20, 40, 60, 80, and 100 μM), 465 (final concentrations 10, 20, 30, 40, and 50 μM). Control wells received vehicle alone.

After 16 h exposure, media were aspirated, cells washed, and TMZ-free media introduced. Plates were incubated for 11 days at 37°C in an atmosphere of 5% CO_2_. Cells were washed (3 × in phosphate buffered saline), fixed with pre-chilled methanol (100%; 20 min), stained with 0.5% methylene blue in 1:1 methanol/H_2_O (v/v) for 10 min, washed thoroughly in dH_2_O and air-dried. Cell colonies containing >50 cells were counted and IC_50_ values were calculated by interpolation.

### Detection of γ-H2AX Foci

HCT116 and T98G cells (2 ×10^5^) were seeded onto coverslips in 6-well plates and incubated overnight, before being exposed to 377 (50 and 100 μM) and 465 (20 and 50 μM) for 3, 6, 9, 12, and 24 h. Cells were fixed with ice cold acetone, rinsed with PBS and blocked with 3% BSA and treated with 0.3% Triton-X in PBS for 30 min at room temperature. Cells were incubated with primary antibody (1° Ab) recognizing phosphorylated H2AX (γH2AX; Cell Signaling; overnight at 4°C), then fluorescence-conjugated Alexa Fluor 488 secondary (2°) Ab (Life technologies; 8 μg/ml diluted in antibody dilution buffer) for 60 min at room temperature protected from light. Nuclei were stained with 0.1 μg/ml DAPI. Cells were observed by immunofluoroscence microscopy (Nikon, Japan; original magnification 100 × ) and images captured.

### Pulsed-Field Gel Electrophoresis

Cells (2 ×10^6^) were seeded onto coverslips in plates and incubated overnight before treatment with 100 μM TMZ, 100 μM 377, and 50 μM 465 for 24 and 48 h. Cells (6 ×10^5^) were collected from each sample and small agarose plugs embedded with cells were prepared. The small plugs were then digested with Proteinase K reaction buffer (10 mM Tris, 20 mM NaCl, 50 mM EDTA, and 1 mg/ml Proteinase K; 50°C, 48 h). The plugs were washed four times in 50 ml of wash buffer (20 mM Tris, pH 8.8, 50 mM EDTA) for 30 min each at room temperature with gentle agitation. DNA fragments in plugs were separated on 1% w/v agarose gels in 0.5 × Tris/borate/ethylenediaminetetraacetic acid (TBE) buffer at 14°C using a CHEF DRII apparatus (Bio-Rad) with 6 V/cm, pulsed from 60 to 120 s for 24 h. The gels were stained with ethidium bromide (EB). Images were recorded using a gel imager (BIOGEN).

### Western Blot Analysis

Cells were lysed in RIPA lysis buffer (25 mM Tris HCl (pH 7.5), 2.5 mM EDTA, 2.5 mM EGTA, 20 mM NaF, 1 mM Na_3_VO_4_, 100 mM NaCl, 20 mM sodium -glycerophosphate, 10 mM sodium pyrophosphate, 0.5% triton X-100) supplemented with a protease inhibitor cocktail (Roche). Cellular proteins (30 μg) were separated by SDS-PAGE, and electro-transferred onto PVDF membranes. Membranes were blocked in Tris-buffered saline (TBS) containing 5% milk and 0.1% Tween-20 at room temperature. Membranes were incubated with 1° Abs (γH2AX, actin, tubulin, PARP, and caspase 3, from Cell Signaling) overnight at 4°C; membranes were washed at room temperature before incubation with 2° Ab (GE) conjugated with horseradish peroxidase for 1 h. All antibodies were diluted according to the manufacturer's recommendations. Detection was performed with Super Signal Chemiluminescent reagent according to the manufacturer's protocol (Tanon, China).

### Alkaline Agarose Gel Electrophoresis

TMZ, 377, and 465 (1, 10, and 20 μM) were mixed with 1.5 μg pEGFP-N1 plasmid in 40 μl buffer (3 mM NaCl, 1 mM Na_3_PO_4_, and 1 mM EDTA, pH 8.0) and reacted at 37°C for 2 h. Then restriction endonuclease BamHI was added to linearize the plasmid, and DNA was precipitated with ethanol. DNA precipitates were dissolved by 1 × alkaline agarose electrophoresis buffer (10 × alkaline agarose electrophoresis buffer: 500 mM NaOH and 10 mM EDTA, pH 8.0). The desired amount of powdered agarose was thawed in a measured amount of water, cooled to ~55°C, and 0.1 × volume of 10 × basic agarose electrophoresis buffer was added. Then 20 μl dissolved DNA was mixed with 4 μl 6 × basic loading buffer (300 mM NaOH, 6 mM EDTA, 18% Ficoll 400, 0.15% bromocresol green, 0.25% xylene cyanol). Samples were loaded and electrophoresed at a maximum of 5 V/cm at 4°C until the bromocresol green had migrated~two-thirds of the gel length. The gels were placed in a neutralizing solution (1.5 M NaCl and 1 mM Tris-HCl, pH 7.6) and soaked for 45 min at room temperature. The gels were stained with EB and final results recorded using a gel imager (BIOGEN).

### Cell Cycle Analysis

Exponentially growing cells were harvested and seeded in 6-well plates (2 ×10^5^ cells/well; 2 ml medium). Cells were incubated overnight, then treated with TMZ (100 μM), 377 (50 and 100 μM), and 465 (20 and 50 μM). Following incubation (24, 48, 72, and 96 h), attached and floating cells were pooled and pelleted by centrifugation (1,200 rpm, at 4°C, 5 min). Pellets were washed (PBS), cells were re-suspended in 0.3 ml hypotonic fluorochrome solution (0.1% sodium citrate, 0.1% Triton X-100, 50μg/ml PI, and 0.1 mg/ml ribonuclease A) and stored overnight at 4°C in the dark. Fluorescence of PI-stained DNA was detected on a BD C6 cytometer and data were analyzed using C6 software.

### Mitochondrial Membrane Potential Assay

Cells (2 ×10^5^) were seeded in 6-well plates and incubated overnight, then cells were treated with 10, 20, and 50 μM 465 for 9 h. The experimental procedure was carried out according to the JC-10 Mitochondrial Membrane Potential Assay Kit instructions (Sangon, China). Cells (5 ×10^5^ per tube) were centrifuged and pellets suspended in 500 μl of JC-10 dye loading solution and incubated for 40 min at room temperature or 37°C in a 5% CO_2_ incubator. JC-10 monomers and J-aggregates were detected by flow cytometry on FL1 and FL2 channels, respectively. Data were analyzed using C6 software.

### Sample Extraction and Purification

Calf thymus DNA (40 μl; 0.2 mg) and 40 μl 377 (0.2 mg) were dissolved in 120 μl NaCl (15 mM) and incubated in 37°C for 24 h. The reaction mixture was then cooled to room temperature, and extracted with 250 μl diethyl ether 4 × with centrifugation. The extracted DNA was precipitated with 100 μl 3 M sodium acetate solution (pH 5.2) and 1 ml ice-cold ethanol. The solution was then put on ice for 30 min and centrifuged at 4°C at 10,000 g for 15 min. The resulting DNA pellet was washed twice with 100 μl ethanol (70%) and dried under vacuum. The dried DNA pellet was re-dissolved in 100 μl 2 mM magnesium acetate solution, then 2 μl 50 units benzonase enzyme were added before incubation at 37°C for 4 h. Nuclease S1 enzyme (10 μl; 200 units) was added, and the solution was further incubated at 37°C for 4 h. Finally, solid-phase extraction (SPE) was carried out for these samples as follows: the cartridges were conditioned with 3 ml methanol and 2 ml 15 mM NaCl (pH 7.4). After sample absorption, cartridges were washed with 3 ml 15 mM NaCl to remove unmodified nucleotides and modified nucleotides were eluted from the cartridges with 2 ml methanol. The solvent was removed in a vacuum centrifuge and the dried residues were re-dissolved in 100 μl purified water.

### HPLC

The LC system consisted of an Agilent (USA) Accela HPLC pumping system, coupled with an Accela Autosampler and Degasser. Chromatographic separation of the DNA-adducts was achieved by reverse phase chromatography and gradient elution. Separation of the DNA-adducts was carried out on a Perfluorophenyl column (Phenomenex Luna, USA), at 25°C. The gradient mobile phase was prepared from 1% ethanoic acid, pH 5.5 (Eluent A) and methanol (Eluent B): gradient program: 0 min 95% A, 0–6 min up to 95% B, 6–8 min 95% B, 8–8.1 min back to 95% A, 8.1–11 min equilibration. The flow rate was 1 ml/min and the injected volume was 5 μl.

### Compound Stability Test

TMZ or 377 (20 μl; 5 mg/ml) were mixed thoroughly with 80 μl pure water to make final concentration of 5.1 mM for TMZ and 4.6 mM for 377 in a 37°C incubator. At different time points (0–48 h), samples were taken for detection, and filtered using a 13 mm 0.2 μm polytetrafluoroethylene (PTFE) needle prior to HPLC detection. The data processing was carried out with Agilent 1260 Infinity.

### UPLC-Q-TOF/MS Analysis

The Agilent 6540 UPLC-Q-TOF / MS (USA) was used with electrospray ionization for DNA adducts qualitative detection. Chromatographic separation of DNA adducts was achieved by reverse phase chromatography and gradient elution. The detection conditions of UPLC were the same as those for HPLC. Analysis was performed on a triple quadrupole time of flight mass spectrometer, fitted with a heated electrospray ionization source Jet Stream operating in the positive ion mode with the following working conditions: dry gas N_2_ temperature of 350°C and flow rate of 9 l/min; nebulizer pressure of 40 psig; capillary voltage of 3,500 V; nozzle voltage of 1,000 V; fragmentor voltage of 135 V; secondary mass spectral collision pool voltage of 20 V. For full MS investgations, scans range from 10 to 500 *m/z* using a scan rate of 10 spetra/s. Finally, data processing was carried out with MassHunter software.

### Statistical Analysis

Statistical significance was determined using Students' *t*-test analyses. *P* ≤ 0.05 was considered significant.

## Results

### Growth Inhibition

The activity of TMZ and analogs 377 and 465 was tested against a pair of isogenic human GBM cell lines: SNB19V (vector control) and SNB19M (stable MGMT transfection). TMZ was active in MGMT negative/low cell lines, with an IC_50_ value of 38.01 μM in SNB19V cells, but inactive in MGMT-overexpressing cells. SNB19M cell line transfected with MGMT demonstrated 13.39-fold inherent resistance to TMZ with a mean IC_50_ value of 508.84 μM. As can be seen in Table 1, C8-imidazolyl 377 showed anticancer activity in SNB19V and SNB19M cells with mean IC_50_ values of 32.96 μM, and 65.92 μM, respectively. Methyl imidazole analog 465 gave IC_50_ values of 14.34 μM and 31.83 μM in SNB19V and SNB19M cells, respectively. In addition, 377 and 465 showed good anticancer activity against the T98G GBM cell line which maintains inherently high MGMT expression, with IC_50_ values of 62.50 μM and 33.09 μM, respectively. Therefore, MGMT expression whilst conferring marked resistance to TMZ, conferred only ~2-fold resistance to 377 and 465. Furthermore, the MMR-deficient HCT116 CRC cell line, resistant to TMZ (IC_50_ values > 500 μM), responded to 377 (IC_50_ value 44.23 μM) and 465 (IC_50_ value 25.37 μM). Consistently, methylimidazole analog 456 demonstrated~twice the potency of imidazolyl analog 377.

**Table 1:** MTT assay IC_50_ values of TMZ and C8-imidazotetrazine analogs 377 and 465 against human cancer cell lines.

**Cell line**	**MGMT**	**MMR**	**IC**_****50****_ **(μM) 3 days**		
			**TMZ**	**377**	**465**
SNB19V	–	Proficient	38.01 ± 16.35	32.96 ± 10.80	14.34 ± 2.92
SNB19M	+	Proficient	508.84 ± 142.32	65.92 ± 7.68	31.83 ± 8.66
HCT116	+	MLH1-	592.88 ± 16.10	44.23 ± 12.72	25.37 ± 2.21
T98G	+	Pro5ficient	286.96 ± 19.61	62.50 ± 6.93	33.09 ± 3.42

*TMZ, 377, and 465 were dissolved in DMSO at a stock solution of 100 mM (stored at −20°C) and diluted in culture medium prior to use. IC_50_ values of new analogs were determined by MTT assay following 3 days of exposure and are shown as mean ± SD of three independent experiments*.

*In vitro*, the antitumor activity of TMZ derivatives was further corroborated by clonogenic assays. As shown in Table 2, compared to SNB19V cells (IC_50_ value 32.2 μM), SNB19M cells demonstrated ~27-fold inherent resistance to TMZ (IC_50_ value 854.65 μM) following 11 days exposure of SNB19V and SNB19M cells to TMZ. Compared to SNB19V cells (IC_50_ value 17.86 μM), SNB19M cells showed > 6-fold resistance to C8-imidazole analog 377 (IC_50_ value > 100 μM). C8-methylimidazole analog 465 exerted potent inhibition of colony formation in SNB19V cells (IC_50_ value 14.34 μM) and moreover, potent anti-clonogenic activity against MGMT-expressing SNB19M cells (IC_50_ 31.83 μM).

**Table 2:** Clonogenic assay IC_50_ values of TMZ and C8-imidazotetrazine analogs 377 and 465 against human cancer cell lines.

**Compound**	**SNB19V**	**SNB19M**	IF^*^
TMZ	32.20 ± 10.35	854.65 ± 142.32	27
377	17.86 ± 7.30	>100	>6
465	14.34 ± 2.92	31.83 ± 8.66	2

IC_50_ values of TMZ and new analogs were determined by colony-formation assay following 11 days incubation after compound treatment (16 h) and are shown as mean ± SD of three independent experiments, IF

* *: SNB19M IC_50_ value/SNB19V IC_50_ value*.

These results indicate that novel imidazolyl and methylimidazole imidazotetrazinones 377 and 465 inhibit the growth of tumor cells irrespective of MGMT and MMR; of note, 465 demonstrated superior activity (~2-fold) against TMZ-resistant cells guiding compound concentration selection in further experiments.

### Generation of DNA Double-Strand Breaks by Imidazotetrazine Analogs

**Figure 2:**
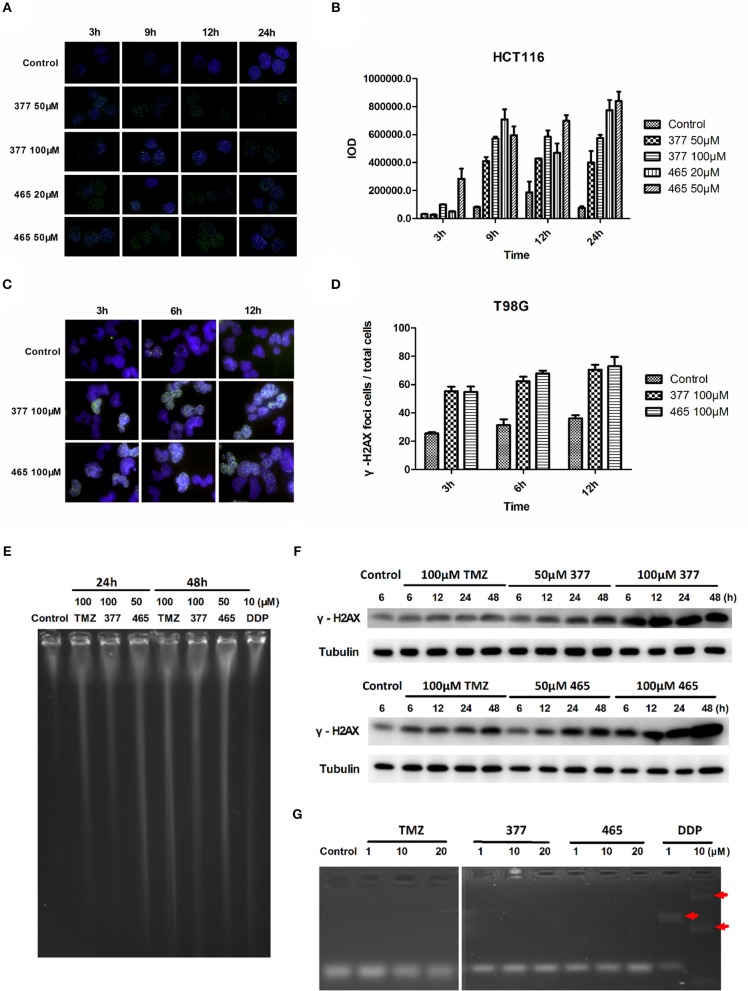
C8-imidazotetrazine analogs 377 and 465 induce cancer cell DNA damage. **(A)** Induction of γH2AX foci in HCT116 cells following exposure to 377 and 465. HCT116 cell was treated with vehicle control, 50 μM 377 and 465 for 3, 9, 12, and 24 h; **(B)** Foci of treated HCT116 cells were quantified by densitometry (Image J software). **(C)** Induction of γH2AX foci in T98G cells. T98G cells were treated with vehicle control, 100 μM 377 and 465 for 3, 6, and 12 h, then immunocytochemically labeled with γH2AX antibody and the secondary antibody Alexa Fluor 488; DNA was counterstained with DAPI. **(D)** γH2AX quantification was performed by the ratio of DNA damaged cells to total T98G cells (cellular foci number > 10 was considered to be damaged cell). **(E)** Pulsed-field gel electrophoresis (PFGE) directly detected DSBs in HCT116 cells. HCT116 cells were treated with TMZ (100 μM), 377 (100 μM), 465 (50 μM), and DDP (10 μM, as a positive control) for 24 and 48 h. **(F)** Detection of total γH2AX expression by Western blot. T98G cells were treated with TMZ, 377, or 465 (50 and 100 μM) for 6, 12, 24, and 48 h. **(G)** Detection of DNA cross-linking by alkaline gel electrophoresis. The double strand DNA was incubated with 1, 10, and 20 μM TMZ, 377, and 465, DDP (cisplatin) (10 μM) was used as a positive control. The arrow marked the cross-linked DNA.

TMZ generates *O6*-MeG adducts which trigger futile cycles of mismatch repair (MMR) in MMR-proficient cells that ultimately lead to lethal DNA double-strand breaks (DSBs). DSBs induce rapid phosphorylation of Ser139 at the carboxy terminus of histone H2AX, namely γH2AX; γH2AX has become the “gold standard” marker of DNA DSBs. In order to determine whether 377 and 465 evoke DNA DSBs, the formation of γH2AX foci was examined in HCT116 cells treated with 377 (50 and 100 μM) and 465 (20 and 50 μM) and visualized by immunofluorescence microscopy following 3, 9, 12, and 24 h exposure. Representative foci in HCT116 cells are shown in Figure 2A and foci quantification of treated cells is shown in Figure 2B. As demonstrated, new analogs 377 (50 and 100 μM) and 465 (20 and 50 μM) induce time- and concentration-dependent DNA DSBs in HCT116 cells. In addition, T98G cells were treated with 100 μM 377 and 465 for 3, 6, and 12 h, cellular γH2AX foci increased time-dependently (Figures 2C,D).

Moreover, pulsed-field gel electrophoresis (PFGE) was adopted to detect genomic DNA DSBs. HCT116 cells were treated with TMZ (100 μM), 377 (100 μM), 465 (50 μM), or DDP (cisplatin, 10 μM; positive control) for 24 and 48 h, and disruption of genomic DNA was detected by PFGE (Figure 2E). TMZ and 377 both induced DSBs in HCT116 cells and the extent of genomic DNA breakage increased with time. Furthermore, global γH2AX expression was examined by Western blot following exposure of T98G cells to TMZ (100 μM), 377 (50 and 100 μM), and 465 (50 and 100 μM) for 6, 12, 24, and 48 h. As shown in Figure 2F, TMZ only slightly increased the expression of γH2AX in T98G cells, whereas, the same concentration of 377 and 465 caused more extensive, time-dependent γH2AX expression. Together these data indicate that new analogs 377 and 465 cause DNA damage in TMZ-resistant cells.

It is known that the TMZ analog mitozolomide results in DNA cross-linking (18), which causes severe and unpredictable bone marrow suppression (19). Therefore, we examined by alkaline gel electrophoresis whether the TMZ derivatives caused DNA cross-links. Double-stranded DNA was treated with 1, 10, and 20 μM TMZ, 377, and 465, DDP (1 and 10 μM) was used as a positive control; as Figure 2G demonstrates, imidazotetrazine analogs TMZ, 377, and 465 did not lead to formation of DNA cross-links.

### Cell Cycle Perturbation by Imidazotetrazine Analogs

**Figure 3:**
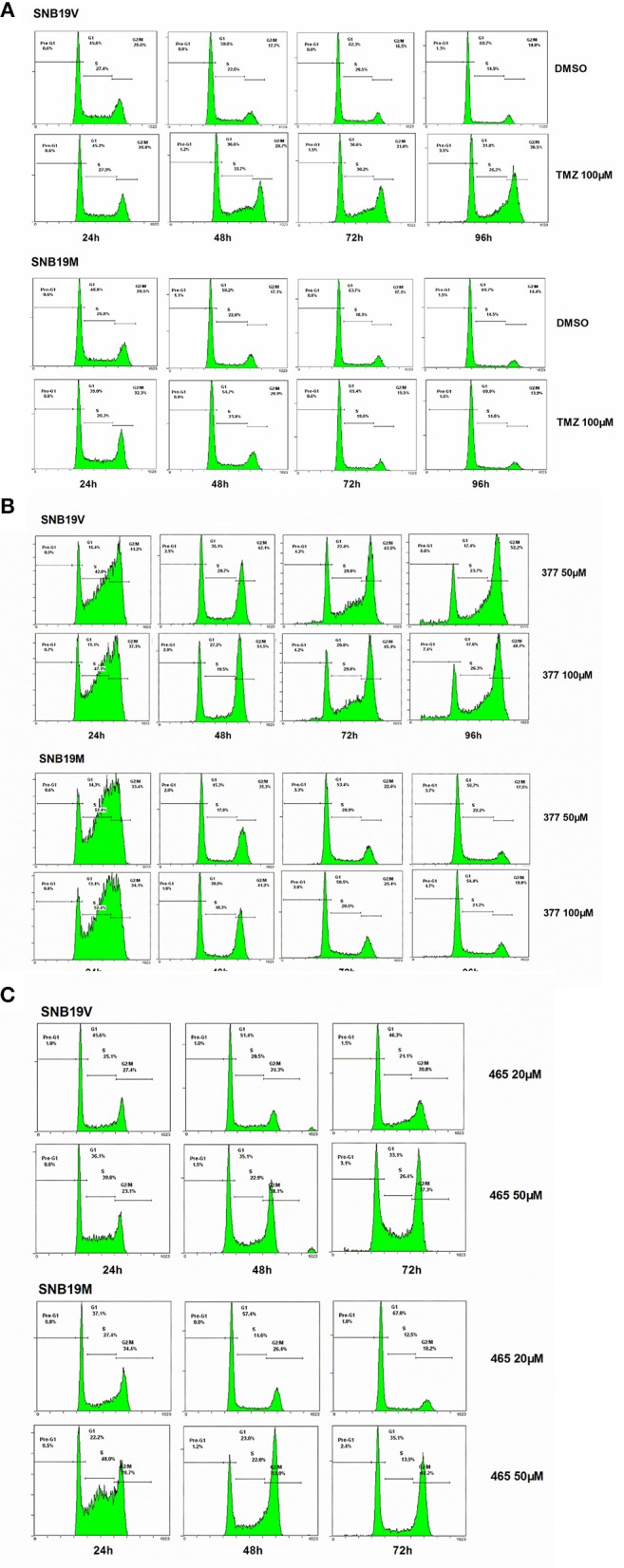
377 and 465 caused cell cycle arrest in SNB19V and SNB19M cells. Representative DNA histograms of SNB19V and SNB19M cells demonstrating the effects of TMZ **(A)**, 377 **(B)**, and 465 **(C)** on cell cycle progression. Following desired exposure periods, cellular DNA was stained with propidium iodide and analyzed by flow cytometry. Control samples were treated with equal volume DMSO. For each sample, 10,000 events were recorded and experiments were performed in triplicate; *n* ≥ 3 independent trials.

Futile DNA repair cycles triggered by TMZ result in cell cycle arrest in the absence of MGMT and the presence of MMR. To compare the effects of TMZ and analogs on cell cycle progression, DNA flow-cytometric analyses of SNB19V and SNB19M cells following exposure to agents (24–96 h) were performed. After 48 h treatment with 100 μM TMZ, 28.7% of the SNB19V cells arrested at G2/M (compared with 17.7% of control cells in G2/M; Figure 3A). Further significant (*P* < 0.01) and prolonged accumulation of DNA in G2/M phases was observed after 72 and 96 h of exposure (31.8 and 48.5%, respectively). In contrast, 100 μM TMZ failed to perturb SNB19M cell cycle, a result attributed to competent MGMT activity (Figure 3A).

Exposure of SNB19V cells to 377 (50 μM) significantly (*P* < 0.001) increased S-G2/M events by > 15% during the first 24 h period, persistent G2/M accumulation was maintained from 48 to 96 h, and a small pre-G1 peak indicative of apoptosis induction emerged; 6.6% pre-G1 phase at 96 h (0.9% pre-G1 in control) (Figure 3B). Compared to SNB19V cells, SNB19M cells also exhibited significant (*P* < 0.001) S-G2/M arrest at 24 h exposure, but the S-G2/M block was transient—reversing ≥48 h (Figure 3B). Cell cycle perturbations caused by 377 (100 μM) in both SNB19V and SNB19M were similar to those elicited by 50 μM 377.

SNB19V and SNB19M cells exposed to 465 (50 μM) revealed obvious (*P* < 0.01) S-G2/M arrest at 24 h. Further significant (*P* < 0.001) and prolonged accumulation of G2/M phase events was observed from 48 to 72 h (38.1 and 37.3% in SNB19V; 53.0 and 48.2% in SNB19M populations, respectively; Figure 3C). The percentage of the cell population in G2/M was higher in SNB19M cells than SNB19V cells, 53.0% compared to 38.1% at 48 h and 48.2% compared to 37.3% at 72 h. Analog 465 (20 μM) minimally perturbed SNB19V and SNB19M cell cycles (Figure 3C). The effect of TMZ derivative 465 on the cycle of MMR-deficient HCT116 cells was also examined. It was evident that events in G2/M accrued at both 48 and 72 h (50 μM 465) while 50 μM TMZ did not interfere with the cell cycle (Figure S1). These data indicate that TMZ analogs 377 and 465 cause cell cycle perturbation in TMZ-resistant cells.

### Induction of Apoptosis by Imidazotetrazine Analogs

**Figure 4:**
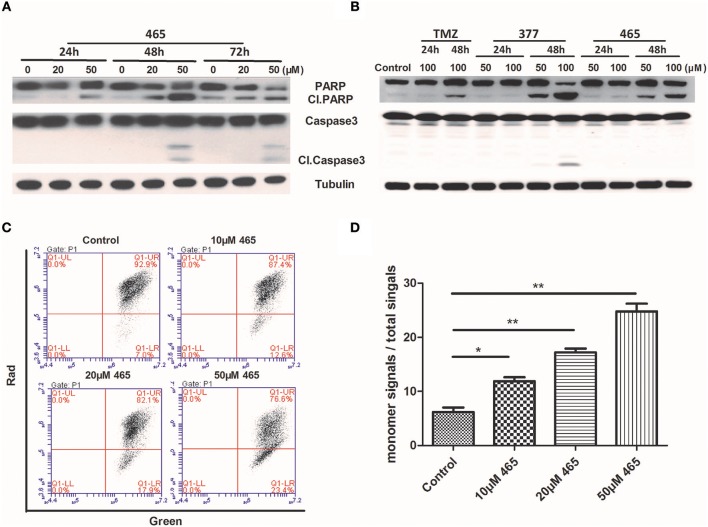
Induction of apoptosis by C8-imidazotetrazine analogs 377 and 465. Detection of apoptosis proteins (PARP and caspase 3) by Western blot in **(A)** HCT116 cells and **(B)** T98G cells following treatment of cells with different concentrations TMZ, 377, or 465 for 24, 48, or/and 72 h. **(C)** HCT116 cells were dye-loaded with JC-10 dye-loading solution along with 10, 20, and 50 μM 465 for 9 h. The fluorescent intensities for both J-aggregates and monomeric forms of JC-10 were measured by flow cytometry using FL1 (green) and FL2 (red) channels. Data were analyzed using C6 software. **(D)** Quantitative assessment of JC-10 monomer signals. (*P < 0.05, **P < 0.01).

In order to further investigate the cellular effects of 377 and 465, we characterized their impact on cell death at the molecular level. Lysates from compound-treated cells were subjected to Western blot analyses for expression of two apoptosis markers, caspase 3 activation, and cleaved PARP. Initially, HCT116 cells were treated with the same concentration (50 μM) of TMZ and its derivatives for 48 h. As shown in Figure S2, we found that only 465 induced PARP cleavage. HCT116 cells were subsequently treated with 20 and 50 μM 465 for 24, 48, and 72 h. 465 induced apoptosis in a time- and concentration-dependent manner: 20 μM 465 analog induced PARP cleavage ≥48 h, however, cleaved PARP was detected in lysates of cells treated with 50 μM 465 ≥24 h (Figure 4A). In addition, 50 μM 465 also led to cleaved caspase (48 and 72 h). In addition, MGMT-overexpressing T98G cells were also exposed to 100 μM TMZ, 50, and 100 μM 377 and 465 for 24 and 48 h. As shown in Figure 4B, neither significant caspase 3 nor PARP cleavage were induced by TMZ, 377, or 465 cells after 24 h. However, cleaved PARP was detected ≥48 h by 50 μM 377 and 465, and 100 μM TMZ. These data indicate that analogs 377 and 465 cause apoptosis in TMZ resistant cells.

To corroborate this conclusion, changes in HCT116 cells mitochondrial membrane potential was determined after exposure of cells to 465 (20). As shown in Figure 4C, when HCT116 cells were treated with 10, 20, and 50 μM 465 for 9 h; the JC-10 monomer signals increased significantly (*P* < 0.05) in a concentration-dependent manner (Figures 4C,D).

### Hydrolysis of Imidazotetrazine Analog 377

**Figure 5:**
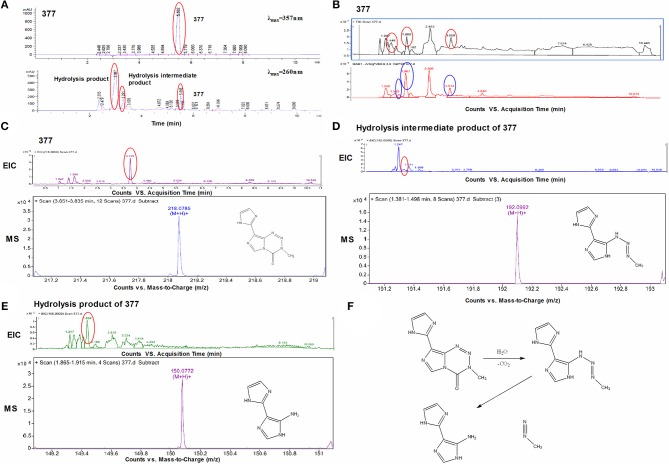
Decomposition of C8-imidazotetrazine analog 377. **(A)** Chromatogram of 377 by HPLC. **(B)** Total ion chromatogram of 377 by UPLC-Q-TOF/MS. **(C)** Mass spectra of 377 (C_8_H_7_N_7_O) at *m/z* 218.0786. **(D)** Mass spectra of intermediate (C_7_H_9_N_7_) at *m/z* 192.0992. **(E)** Mass spectra of metabolite (C_6_H_7_N_5_) at *m/z* 150.0772. **(F)** Reaction scheme for the decomposition of 377 in H_2_O.

Under physiological conditions, TMZ is able to hydrolyze to form MTIC which rapidly breaks down to form diazomethane and 5-aminoimidazole-4-carboxamide (AIC); diazomethane causes methylation of DNA, leading to cell death. We hypothesize that 377 could ring-open and alkylate DNA like TMZ, resulting in DNA damage to tumor cells and subsequent apoptosis. Analog 377 was dissolved in water, incubated at 37°C and analyzed by HPLC. As shown in Figure 5A, three peaks were observed at retention times *t*_R_ = 2.12 min, *t*_R_ = 2.43 min, and *t*_R_ = 4.43 min. The hydrolyzate of 377 was then identified using a highly sensitive UPLC-Q-TOF/MS. As shown in Figure 5B, the absorption peaks can be detected in the total ion flow chromatogram. They were the intermediate, metabolite and absorption peaks of 377.The structures were assigned on the basis of UPLC-Q-TOF/MS data. The absorption peak of 377 had a retention time of 3.568 min and an ion [M+H]^+^at *m/z* 218.0786, indicating that the molecular formula was C_8_H_7_N_7_O. The intermediate had a retention time of 1.448 min and an ion [M+H]^+^at *m/z* 192.0992, indicating that the molecular formula was C_7_H_9_N_7_. The metabolite had a retention time of 1.999 min and exhibited an ion [M+H]^+^at *m/z* 150.0772, indicating that the molecular formula was C_6_H_7_N_5_ (Figures 5C–E). Therefore, we speculate that (similar to TMZ) 377 can undergo hydrolysis. These data indicate that 377 is rapidly hydrolyzed to form the intermediate 5-(3-methyltriazen-1-yl) imidazole-4-imidazole under physiological conditions, and then undergoes further hydrolysis to form 5-aminoimidazole-4-imidazole and a diazonium ion (Figure 5F).

### Stability of Imidazotetrazine Analog 377

**Figure 6:**
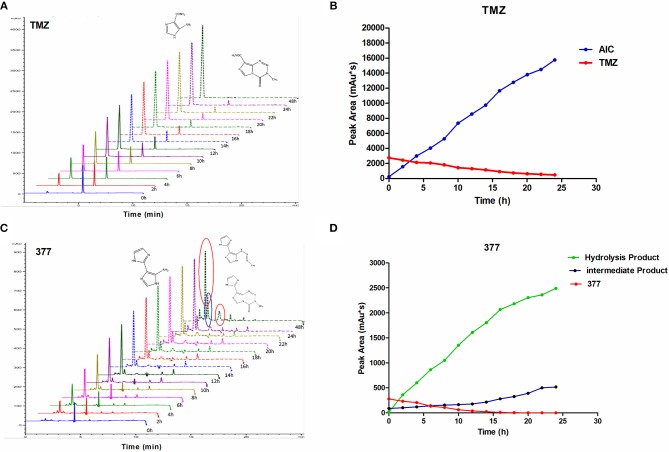
The stability of TMZ and 377. **(A)** The UV absorption spectra of TMZ, TMZ was incubated at 37°C for different times. **(B)** Quantitative peak area of TMZ. **(C)** The UV absorption spectra of 377, 377 was incubated at 37°C for different times. **(D)** Quantitative peak area of 377.

The stability of 377 in H_2_O was monitored by HPLC. TMZ or 377 solutions (1 mg/ml) were incubated at 37°C for 0–48 h. As shown in Figure 6A, TMZ was readily hydrolyzed at 37°C, and almost completely hydrolyzed to form AIC at 48 h. The intermediate product MTIC was very unstable and the absorption of MTIC could not be detected by UV detector. The peak area of TMZ decreased from 2,758 mAU^*^s to 475 mAU^*^s at 37°C between 0 and 24 h, reaching 50% after 10 h. The absorption peak of TMZ could not be detected at 48 h, but the absorption peak of AIC increased with time (Figure 6B). However, the peak area of 377 at 37°C decreased from 279 mAU^*^s to 0 mAU^*^s over 20 h, reducing to 50% after 6 h. The peak of 377 could not be observed at 20 h (Figures 6C,D), correspondingly, the peak areas of the intermediate and hydrolysate accumulated over time (0–20 h): from 87 mAU^*^s to 518 mAU^*^s and from 21 to 2,491 mAU^*^s, respectively. These dat suggest that 377 hydrolyzes more rapidly than TMZ, and the intermediate is more stable than that of TMZ (MTIC).

### DNA Adducts of Imidazotetrazine Analog 377

**Figure 7:**
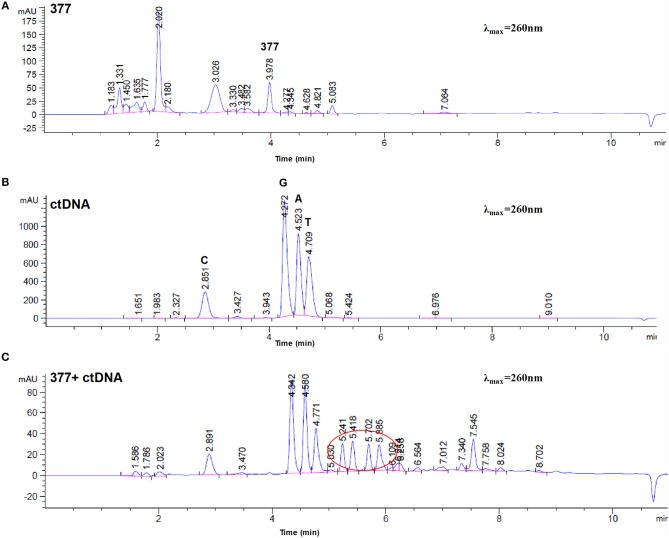
Chromatogram of 377 with ctDNA by HPLC. **(A)** Chromatogram of 377. **(B)** Chromatogram of ctDNA. **(C)** Chromatogram of 377 with ctDNA.

**Figure 8:**
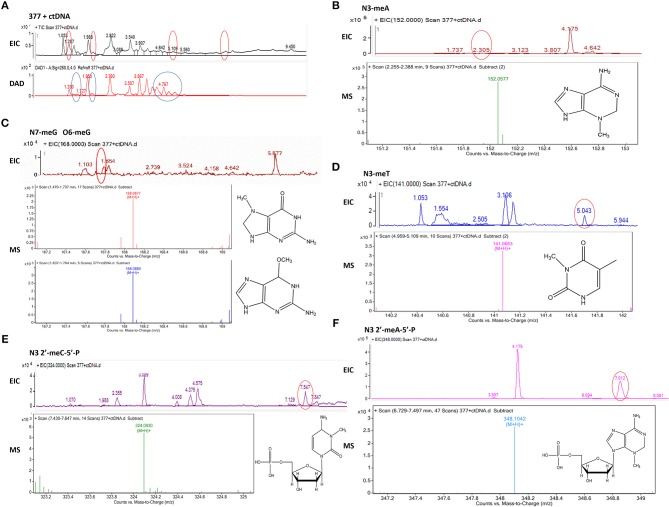
Mass spectra of 377. The incubation of 377 with ctDNA for 24 h followed by enzymatically digestion with benzonase and nuclease S1. **(A)** Total ion chromatogram of 377 with ctDNA. **(B)** Mass spectra of N3-methyladenine (C_6_H_9_N_5_) at m/z 152.0577. **(C)** Mass spectra of N7-methylguanine and O6-methylguanine (C_6_H_9_N_5_O) at *m/z* 168.0877 and 168.087. **(D)** Mass spectra of N3-methylthymine (C_6_H_8_N_2_O_2_) at *m/z* 141.0663 141. **(E)** Mass spectra of N3-methylcytidine deoxynucleotides (C_6_H_18_N_3_O_7_P) at *m/z* 324.0930. **(F)** Mass spectra of N3-methyladenine deoxynucleotides (C_11_H_18_N_5_O_6_P) at *m/z* 348.1042.

To investigate DNA adducts of 377, calf thymus DNA (ctDNA) was incubated with 377 *in vitro* and the resulting modifications were enzymatically cleaved with benzonase and nuclease S1. Compared with the chromatograms of 377 and ctDNA alone, four new absorption peaks were observed at retention times *t*_R_ = 5.241, *t*_R_ = 5.418, *t*_R_ = 5.702, and *t*_R_ = 5.885 by HPLC analysis (Figures 7A–C). We speculated that DNA adducts may be formed. As described above and shown in Figure 5F, 377 can ring-open to form an intermediate which then further breaks down to a reactive diazonium, we posit that the diazonium will alkylate DNA. Therefore, UPLC-Q-TOF/MS was used to analyze the structures of DNA adducts. As shown in Figure 8A, new absorption peaks in the total ion current profile and the UV absorption pattern are apparent.

Upon analysis, the following deoxynucleotide adducts were identified: *N3*-MeA (*t*_R_ = 2.305 min), [M+H]^+^ ion at m/z 152.0577, molecular formula C_6_H_9_N_5_; *N7*-MeG (*t*_R_ = 1.654 min), [M+H]^+^ ion at m/z 168.0877, molecular formula C_6_H_9_N_5_O, which gave rise to a fragment ion at m/z 151.3526 and showed the loss of a –CH_3_; *O6*-MeG (*t*_R_ = 1.654 min), [M+H]^+^ ion at m/z 168.0880, molecular formula C_6_H_9_N_5_O–gave rise to a weak fragment ion at m/z 151.2436 but demonstrated no loss of –CH_3_, thus is the basis for the distiction between *N7*-MeG and *O6*-MeG; *N3*-MeT (*t*_R_ = 5.043 min), [M+H]^+^ ion at m/z 141.0663, molecular formula C_6_H_8_N_2_O_2_; *N3*-MeC (*t*_R_ = 7.547 min), [M+H]^+^ ion at m/z 324.0930, molecular formula C_6_H_18_N_3_O_7_P; *N3*-MeA (*t*_R_ = 7.012 min), [M+H]^+^ ion at m/z 348.1042, molecular formula C_11_H_18_N_5_O_6_P (Figures 8B–F).

## Discussion

Temozolomide plays a key role in GBM therapy; indeed, radiotherapy, and concomitant and adjuvant chemotherapy with temozolomide is the standard of care for this devastating malignancy. TMZ has also been used in the treatment of other cancers (e.g., malignant melanoma) (21), but the ultimate benefit is limited by inherent, or emergence of resistance. Expression of the DNA repair protein MGMT accounts for TMZ resistance; inactivation or down-regulation of MMR leads to acquired tolerance to TMZ-induced lesions (2, 7) and moreover, TMZ exposure can promote a hypermutator phenotype (22). Therefore, developing new TMZ derivatives which can overcome TMZ-resistance may provide significant therapeutic advantage.

A variety of TMZ derivatives has been previously reported (13–17, 23–28). The most common modification sites for these analogs are at the N3 or C8 positions of TMZ. Among them, N3-modified TMZ analogs can be divided into two categories: one class of derivatives involves incorporation of groups to link two TMZ molecules through the N3 site, for example, derivatives DP68 are two TMZ molecules linked together by methylaniline. DP68 binds two DNA strands together by hydrolysis to cross-link the DNA and thus kill the cells (23). Another class of analogs results from replacement of the methyl group at the N3 position with substituents, such as chloromethyl or propargyl. These derivatives allow the new group to alkylate DNA, thereby exerting a cytotoxic effect (13, 14). In addition, C8-modified TMZ analogs can also be divided into two categories: one class may be derived through conjugation of two TMZ molecules through the C8 position. For example, the analog 2T-P400 links two TMZs by polyethylene glycol, thereby enhancing the solubility and stability of TMZ (24). Another type of derivative may be formed following replacement of the C8 carboxamide group of TMZ by another group. For instance, NEO212 is a covalent bond of perillyl alcohol to the C8 site of TMZ, thereby enhancing the efficacy of TMZ (25–27), while TMZ hexyl ester which incorporates a hexyl ester bound to the C8 site of TMZ can improve TMZ skin delivery and antitumor potency (28). In addition, other C8-substituted derivatives such as 8-cyano-imidazotetrazine and 8-thiotemozolomide have been developed, but their antitumor mechanism(s) remain to be defined (17).

In this study, the anti-cancer activity of two novel C8-substituted TMZ analogs C8 imidazolyl (377) and C8 methyl imidazole (465) has been evaluated. Isogenic GBM cell lines, SNB19V (vector control), and SNB19M (stable MGMT transfection), were used to detect the activity of TMZ and its derivatives. TMZ is active in the MGMT-low cell line, with a mean IC_50_ value of 38.01 μM, but inactive in MGMT-overexpressing SNB19M cells, with a mean IC_50_ value of 508.84 μM. SNB19M cells overexpressing MGMT revealed 13-fold resistance to TMZ (Table 1). However, TMZ analogs 377 and 465 demonstrate activity in SNB19 GBM cell lines, irrespective of MGMT activity. Moreover, relative to TMZ, 377, and 465 show greater potency (~5–24-fold) in MGMT overexpressing glioma T98G and MMR deficient CRC HCT116 cells.

These results may indicate that C8 imidazolyl or methylimidazole confer properties such as greater stability or more efficient delivery than the carboxamide on the imidazotetrazine. Intriguingly, in clonogenic assays, after brief (16 h) exposure of cells to inidazotetrazine analogs, we found that the IC_50_ value of 377 against SNB19M cells was at least 6-fold greater than that in SNB19V cells, while the activity of 465 against SNB19M (compared with SNB19V) cells was only 2-fold greater (Table 2). These data suggest that the lesions imparted by 377 can be repaired by MGMT over time, whereas inclusion of a methyl group within the C8 imidazolyl (465), imparts structural changes that lead to more stable DNA adducts, less susceptible to MGMT repair.

TMZ exerts cytotoxicity primarily through *O6*-MeG, which triggers ineffective cycles of MMR, stalled replication forks and lethal DNA double-strand breaks, leading to cell death by autophagy or apoptosis (2, 4). We found through immunofluorescence, that new analogs 377 and 465 can induce DSB in T98G and HCT116 cells after 6 h treatments (Figures 2A,C). Further study found that TMZ and analogs could induce DSBs in HCT116 and T98G cells. The lower concentration of 465 (50 μM) in HCTl16 cells can result in DNA damage equal to, or greater than that inflicted by the higher concentration (100 μM) of TMZ and 377 (Figure 2D). γH2AX expression in T98G cells was also examined; compared with TMZ, the same concentration of 377 and 465 can upregulate the expression of γH2AX (Figure 2E). Moreover, we found that unlike the TMZ analog mitozolomide, TMZ derivatives 377 and 465 did not produce DNA cross-links (Figure 2F) that can cause serious side effects such as myelosuppression. These results indicate that analogs 377 and 465 can evoke DNA damage in TMZ-resistant cells without leading to DNA cross-linking. We found that TMZ analogs 377 and 465 can lead to G2/M arrest irrespective of MGMT status, and 465 had a greater effect on the cell cycle of MGMT highly-expressing SNB19M cells (Figure 3). Analog 465 also caused G2/M block in HCT116 cells (Figure S1). In addition, combined evidence strongly suggests that C8 analogs 377 and 454 induce apoptotic cell death in TMZ-resistant (MGMT+; MMR-deficient) cells evidenced by sub-G1 cell cycle events (Figure 3), PARP cleavage, caspase activation, and enhanced JC-10 monomer detection (Figure 4). Mitochondrial membrane potential changes indicate that TMZ derivatives may lead to apoptosis dependent on mitochondrial pathways. Together, these data demonstrate that novel C8-substitured TMZ analogs can circumvent TMZ resistance in CRC and GBM cells.

Experiments were undertaken to determine mechanistic activation of C8 analogs, and whether (like TMZ) they act as prodrug vehicles ultimately delivering a toxic methyl lesion to *O6*-guanine. We established that TMZ analog 377 ring-opens and is rapidly hydrolyzed under physiological conditions to form the intermediate 5-(3-methyltriazen-1-yl) imidazole-4-imidazole (MTII), MTII possesses markedly greater stability than MTIC (the hydrolysis intermediate of TMZ), producing 5-aminoimidazole-4-imidazole and diazonium (Figure 5F). The reactive diazonium produces methylated DNA lesions at multiple sites including *N3*-MeA, *N7*-MeG, and *O6*-MeG (Figures 8B,C). Thus, as shown in (Figures 6A–C), 377 hydrolysis is faster than TMZ, and the intermediate is more stable which may explain why 377 exerts activity in TMZ-tolerant or -resistant HCT116 and T98G cells. However, long term, the damage accrued from exposure of cells to 377 can be repaired by MGMT and 377 activity is therefore attenuated (Table 2, Figure 3B), as supported by clonogenic assays. Therefore, replacement of C8 carboxamide by imidazolyl (377) and methyl imidazole (465) may enhance stability of intermediates which might induce DNA damage and apoptosis in MGMT overexpressing and MMR deficient CRC cells. Unfortunately, analyses of stability, and DNA adduct formation by analog 465 was thwarted by precipitation which occurred during incubation of 465 with ctDNA.

In summary, novel C8-substituted TMZ analogs 377 and 465 elicit *in vitro* antitumor activity irrespective of MGMT and MMR status. DNA DSBs were generated in MGMT-overexpressing glioma SNB19M and T98G cells and MMR deficient CRC HCT116 cells. TMZ derivatives perturbed and arrested SNB19V and SNB19M cell cycle progression. Moreover, compared to TMZ, analogs 377, and 465 were able to induce more extensive apoptosis in HCT116 and T98G cells. Similar to TMZ, analog 377 undergoes to ring opening and hydrolysis leading to DNA adducts formation which likely underlies the anticancer effects. These analogs may offer potential for TMZ-resistant GBM and broader spectrum malignancies.

## Data Availability

All datasets generated for this study are included in the manuscript and/or the Supplementary Files.

## Author Contributions

JZ designed the experiment. ZY, DW, and XD performed the experiment and analyzed data. ZY wrote the manuscript. JZ, MS, and TB reviewed and revised the manuscript. YL provided technical and material support.

## Contribution to the Field Statement

Temozolomide (TMZ) is the standard of care chemotherapeutic agent used in the treatment of glioblastoma multiforme, however, the intrinsic and acquired resistance limit its clinic application. Developing the new derivatives that can overcome DNA repair is necessary. We synthesized a series of new analogs with modifications at C-8 and N-3 positions, and investigated their activity and mechanism of action. It was found some compounds exhibit anticancer activity superior to TMZ, irrespective of MGMT and MMR.

### Conflict of Interest Statement

The authors declare that the research was conducted in the absence of any commercial or financial relationships that could be construed as a potential conflict of interest.
