# Supplementary material for: C8-Substituted Imidazotetrazine Analogs Overcome Temozolomide Resistance by Inducing DNA Adducts and DNA Damage
Source: Front Oncol. 2019 Jun 11;9:485. doi: 10.3389/fonc.2019.00485 (PMC6584802; doi:10.3389/fonc.2019.00485)
Supplement: Supplementary file 1 [file Data_Sheet_1.docx]

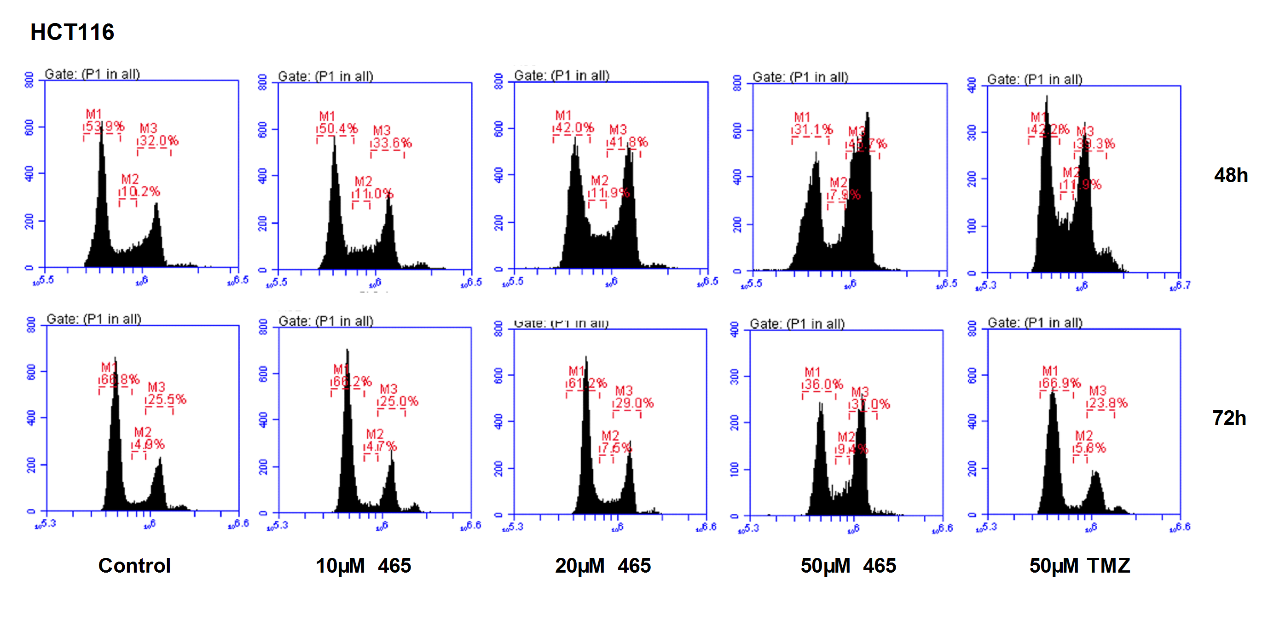


**Figure. S1.** HCT116 cells were treated with 10, 20 and 50 µM 465 for 48 and 72 h, fixed, stained with propidium iodide, and analyzed by flow cytometry.


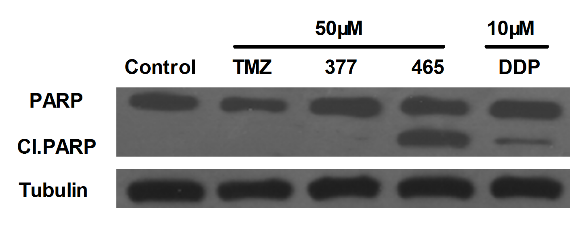


**Figure. S2.** Detection of PARP expression by Western blot in HCT116 lysates following treatment of cells with TMZ (50 μM), 377 (50 μM), 465 (50 μM) and DDP (10μM, as a positive control) for 48h.
